# Revisiting Fold-Change Calculation: Preference for Median or Geometric Mean over Arithmetic Mean-Based Methods

**DOI:** 10.3390/biomedicines12081639

**Published:** 2024-07-23

**Authors:** Jörn Lötsch, Dario Kringel, Alfred Ultsch

**Affiliations:** 1 Institute of Clinical Pharmacology, Goethe University, Theodor Stern Kai 7, 60590 Frankfurt am Main, Germany; 2 Fraunhofer Institute for Translational Medicine and Pharmacology ITMP, Theodor-Stern-Kai 7, 60596 Frankfurt am Main, Germany; 3 Faculty of Medicine, University of Helsinki, 00029 Helsinki, Finland; 4 DataBionics Research Group, University of Marburg, Hans-Meerwein-Straße, 35032 Marburg, Germany

**Keywords:** data science, artificial intelligence, differential expression, omics

## Abstract

**Background:** Fold change is a common metric in biomedical research for quantifying group differences in omics variables. However, inconsistent calculation methods and inadequate reporting lead to discrepancies in results. This study evaluated various fold-change calculation methods aiming at a recommendation of a preferred approach. **Methods:** The primary distinction in fold-change calculations lies in defining group expected values for log ratio computation. To challenge method interchangeability in a “stress test” scenario, we generated diverse artificial data sets with varying distributions (identity, uniform, normal, log-normal, and a mixture of these) and compared calculated fold-changes to known values. Additionally, we analyzed a multi-omics biomedical data set to estimate to what extent the findings apply to real-world data. **Results:** Using arithmetic means as expected values for treatment and reference groups yielded inaccurate fold-change values more frequently than other methods, particularly when subgroup distributions and/or standard deviations differed significantly. **Conclusions:** The arithmetic mean method, often perceived as standard or picked without considering alternatives, is inferior to other definitions of the group expected value. Methods using median, geometric mean, or paired fold-change combinations are more robust against violations of equal variances or dissimilar group distributions. Adhering to methods less sensitive to data distribution without trade-offs and accurately reporting calculation methods in scientific reports is a reasonable practice to ensure correct interpretation and reproducibility.

## 1. Introduction

**Figure 1:**
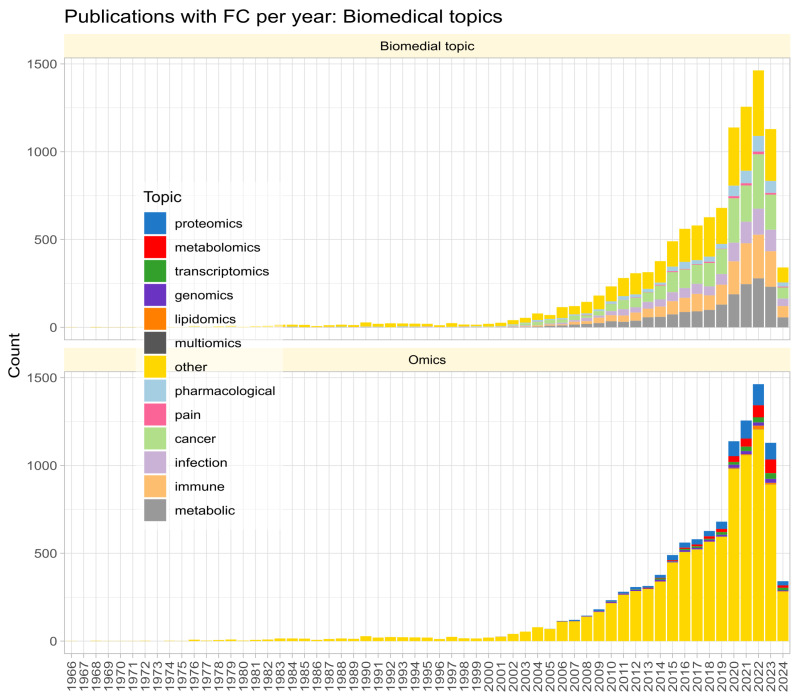
Stacked bar chart of the number of publications per year according to a query of the PubMed database for “(“fold change” NOT (review[PT])” on 23 March 2024. The top panel shows the biomedical topic according to an article categorization of the most frequent biomedical contexts of fold change mention. The lower part shows the same for the term “omics” in the titles, keywords, or abstracts of the hits.

Fold change (FC) is widely used in biomedical research to quantify the magnitude of group differences in omics variables, initially mainly in gene expression studies but nowadays adopted in other “omics” fields and even non-omics research, as evidenced by a PubMed search for “fold change”, where the term was found to be associated with a variety of fields beyond gene expression studies (Figure 1). FC provides a measure for the crucial step in omics data analysis of selecting subsets of genes or other variables of interest from the initial set of variables, along with other methods such as unusual ratio, univariate testing with α-correction for multiple experiments, analysis of variance, and noise sampling methods, comparatively reviewed in [1].

Fold change provides an intuitive measure of the magnitude of the difference between groups. Using ${log}_{2}$ allows direct interpretation of how many times, in multiples of 2, the marker expression was greater or less in the treatment group, and the number of folds by which the expression changes is equal for both up- and down-regulation, with the direction indicated by the sign, e.g., ${log}_{2}\left(3\right)=1.58$ and ${log}_{2}\left(\frac{1}{3}\right)=-1.58$. The term differential expression denotes that the average expression level of a measure in one group is larger or smaller than that in another group [2]. Fold change is often visualized in “volcano plots” [2] using a $-log_{10}$ (*p*_value) on the *y*-axis and log_2_-ratio of signals between treatment and reference groups on the *x*-axis.

Fold-change calculation can significantly influence the interpretation of results and subsequent decision-making processes in biomedical research. Gene lists from microarray studies generated by fold-change ranking were more reproducible than those obtained by *t*-test *p*-value or other significance analyses [3,4], and fold-change is a potential criterion for uni-variate feature selection [5], alone or as a complement to other machine learning-based methods for omics analysis [6].

However, the exact method of calculating fold change is often not reported in scientific publications, although several methods exist. The definition of “average” (such as arithmetic or geometric mean) to quantify group expression levels can lead to inconsistencies. Despite previous studies downplaying the consequences [2,3,7], it seems crucial to carefully choose the most robust method against potential violations of standard assumptions about the data distribution and variance. This report reevaluates the influence of various fold-change calculation methods on fold-change values and aims to recommend a preferred approach.

## 2. Methods

### 2.1. Retrieval of Fold-Change Reporting in Biomedical Publications

On 23 March 2024, a PubMed (https://pubmed.ncbi.nlm.nih.gov/ (accessed on 21 July 2024)) search was performed using the query “(“fold change” NOT (review[PT])”. The R package “easyPubMed” (https://cran.r-project.org/package=easyPubMed [8] (accessed on 21 July 2024)) was used to retrieve details of the papers, including titles, abstracts, and publication years. To identify the main topics where fold-change reporting is common, words in the abstracts were filtered against generic text using the R package ‘PubMedWordcloud’ (https://cran.r-project.org/package=PubMedWordcloud [9] (accessed on 21 July 2024)). Recursive cABC analysis [10]), an item categorization technique, was then applied to the frequency of the remaining words using the R library “ABCanalysis” (https://cran.r-project.org/package=ABCanalysis [11] (accessed on 21 July 2024)). Biomedical domain experts identified biomedical topics based on the relevant terms. The type of omics research covered was determined by applying another cABC analysis to the occurrences of words containing the substring “omics”.

### 2.2. Common Basic Variants of the Fold-Change Calculation

There are numerous descriptions of fold-change calculation, such as in [12]. The calculation is based on the log ratio between the treatment and reference groups of a biological signal found in a data set, i.e., the values of a variable of interest. One common way of calculating the signal log ratio is:

(1) $$logRatio={log}_{2}\left(\frac{E_{b,i}}{E_{a,i}}\right)={log}_{2}\left(E_{b,i}\right)-{log}_{2}\left(E_{a,i}\right)$$

where $E_{a,i}$ and $E_{b,i}$ are the positive, non-zero expected values of variable *i* measured under two different conditions a and b, such as before (*a*) and after (*b*) a treatment, or control (*a*) versus patient (*b*). From the log ratio, the value of fold change, FC, can be obtained by simple maths, for example as given in [12] (Equation (2), first curly brackets), or with an alternative shorter expression (Equation (2), second curly brackets):

(2) $$FC=\left\{\begin{array}{c} 2^{logRatio}\;if\;logRatio\geq 0\\ {-2}^{-logRatio}\;otherwise \end{array}\right\}=\left\{\begin{array}{c} 2^{\left|logRatio\right|}•sign\left(logRatio\right) \end{array}\right\}$$

#### 2.2.1. Definition of Group Average from the Untransformed Data

There are several ways to define the expected values $E_{i}$ for a variable. Often, the arithmetic mean is used and then Equation (1) becomes

(3) $$logRatio={log}_{2}\left(\frac{\bar{b_{i}}}{\bar{a_{i}}}\right)={log}_{2}\left(\bar{b_{i}}\right)-{log}_{2}\left(\bar{a_{i}}\right)$$

where the horizontal line placed over the variable, i.e., $\bar{v}$, denotes the arithmetic mean of variable *v*.

#### 2.2.2. Definition of Group Average from Transformed Data

For log-normally distributed data, the geometric mean serves as a more appropriate measure of central tendency compared to the arithmetic mean. The geometric mean effectively captures the expected value for positive log-normal distributed data, and its application in omics studies has been recommended [13]. It is calculated as the *n*th root of the product of *n* items *x*, i.e., $\sqrt[{n}]{\prod_{i=1}^{n}x_{i}}$, which in the log domain becomes arithmetic mean in log scale, i.e., ${E_{x}=Log}^{\left(\frac{1}{n}\sum_{i=1}^{n}\left(\log_{log}x_{i}\;\right)\right)}={Log}^{\bar{\log x_{i}\;}}$. When also using 2 as the base of logarithm for calculating the log ratio for fold change as in Equation (1), the log ratio is then

(4) $${logRatio=log}_{2}\left(\frac{2^{\bar{\log_{2}b_{i}\;}}}{2^{\bar{\log_{2}a_{i}\;}}}\right)={log}_{2}\left(2^{\bar{\log_{2}b_{i}\;}-\bar{\log_{2}a_{i}\;}}\right)=\bar{\log_{2}b_{i}\;}-\bar{\log_{2}a_{i}\;}$$

denoting the difference of the means of the logs of the two groups *b* and *a*.

Both calculation variants (i.e., calculating fold change via the log of the means as in Equation (3) or calculating it via the mean of the logs as in Equation (4)) are in use. However, for log-normal distributed data, the log of the mean is in general not equal to the mean of the log-transformed data, i.e., ${log}_{2}\left(\bar{x}\right)\;\neq \bar{{log}_{2}\left(x\right)}$ and $FC\neq {FC}^{\prime }$ for the values of fold-change obtained via the log ratios according to Equation (3) or Equation (4), respectively. Another measure of central tendency is the median, which is usually unaffected by the above discrepancy and was used, for example, in [14].

#### 2.2.3. Pairwise Test/Reference Ratio Calculation

As an alternative approach, initially circumventing the necessity of defining the type of group average to be used, fold-change can be calculated by taking the ratio of each paired value from variable *b* and variable *a*. Let $A=\;\left\{a_{1},a_{2},\ldots ,a_{n}\right\}$ be a vector of length $n_{a}$ and $B=\;\left\{b_{1},b_{2},\ldots ,b_{n}\right\}$ be a vector of length $n_{b}$. Then, for all possible combinations of an element of *A* with an element of *B*, expressed as pairsab=a,ba,baϵA,bϵBaϵA,bϵB, the log ratio can be calculated as

(5) $$logRatio=\bar{{log}_{2}\left(\frac{b_{i}}{a_{j}}\right)}\;for\;\left(\left(a_{j},b_{i}\right)\epsilon \;{pairs}_{ab}\right)$$

The pairs are either given if the experimental design involves related samples (e.g., before and after measurements), or they can be created by pairing each case in Group 1 with each case in Group 2 in all possible combinations.

### 2.3. Definition of an Error Measure for Deviations in Fold-Change Calculations

Comparative evaluations were mainly performed on artificial data sets where the true values of the fold changes were known at the time of data generation. Therefore, an error measure for fold-change estimates could be defined as

(6) $${Error}_{FC}\;=\;sign\left({log}_{2}\left({FC}_{calculated}\right)\right)•2^{|{log}_{2}\left({FC}_{calculated}\right)|}\;-sign\left({log}_{2}\left({FC}_{true}\right)\right)•2^{|{log}_{2}\left({FC}_{true}\right)|}$$

### 2.4. Comparative Evaluation of Common Calculation Methods

Evaluations were coded in the R language [15] using the R software package [16], version 4.4.0 for Linux (https://CRAN.R-project.org/ (accessed on 21 July 2024)), and in the matrix laboratory language using MATLAB (version 23.2.0.2485118 (R2023b)) and run on an AMD Ryzen Threadripper 3970X (Advanced Micro Devices, Inc., Santa Clara, CA, USA) desktop computer and an Intel^®^ (Intel Corporation, Santa Clara, CA, USA) Core^TM^ i7-13700H notebook computer, both running on Ubuntu Linux 22.04.4 LTS (Canonical, London, UK). Figures were created using the R libraries “ggplot2” (https://cran.r-project.org/package=ggplot2 [17] (accessed on 21 July 2024)) “ComplexHeatmap” https://www.bioconductor.org/packages/ComplexHeatmap/ [18] (accessed on 21 July 2024), “ggthemes” (https://cran.r-project.org/package=ggthemes [19] (accessed on 21 July 2024)), “GGally” (https://cran.r-project.org/package=GGally [20] (accessed on 21 July 2024) and “ggforce” (https://cran.r-project.org/package=ggforce [21] (accessed on 21 July 2024). Referencing of R packages used for data analyses and visualizations in this report follows published advice on good software citation practice [22]). The equations used in the experiments are summarized in Table 1, along with the abbreviations or acronyms of the methods used in this report.

#### 2.4.1. Evaluation of the Role of the Data Distribution for the Correct Calculation of FC

Fold-change calculations were evaluated on data generated to represent different distributions, including normal and log-normal, as well as identity, uniform, and mixed, where the latter is a random mixture of the four former distributions (Data set # 1; Table 2). Two vectors with different standard deviations of the sample sizes of *a* and *b* were performed using different calculation methods of FC.

#### 2.4.2. Evaluation of the Role of Variance Equality for the Correct Calculation of FC

The above experiments highlighted specific problems with the log-normal distribution for certain variants of the FC computation. Given the frequent log-normal distribution of biological data sets, including non-omics data such as psychophysical measurements [23] and many others, this was brought into focus.

The effect of different values of FC and different standard deviations of treatment ($s_{b}$) and reference ($s_{a}$)) on the accuracy of fold-change recovery was explored in log-normally distributed data (Data set # 2; Table 2). Across a wide range of simulated scenarios, with values of $FC=\left[0.1,\ldots ,6\right]$, $\frac{s_{b}}{s_{a}}=\left[0.1,0.5,1,2,4,8\right]$, and $s_{a}=\left[0.1,1\right]$, the errors in fold-change estimates were quantified according to Equation (6).

#### 2.4.3. Evaluation of the Relationship of FC Calculation to Statistical Outcomes

The two components of a volcano plot, fold change (FC), respectively, the log ratio of treatment and reference and the statistical significance ($-\log_{10}p\;$), were further evaluated using a simulated data set #3 Table 2 containing variables with either normal or log-normal distributions. This data set comprised 99 pairs of vectors, *a* (reference) and *b* (treatment), generated by randomly drawing values for means *m*, standard deviations $s_{a}$ and $s_{b}$, and values of FC from predefined ranges. Different combinations of $Ratio=\left[\frac{1}{3},\ldots ,3\right]$ with different standard deviations $m_{log-normal}=\;U_{1}\left(30,40\right),\;s_{a,b,log-normal}=U_{2}\left(1,5\right),$ $m_{normal}=\;U_{1}\left(500,600\right),\;s_{a,b,normal}=U_{2}\left(20,200\right)$.

Experiments were conducted on these 99 pairs of vectors *a* and *b*, employing different calculations for FC (mean of logs, log of means, paired approach) in combination with both nonparametric (Wilcoxon-Mann-Whitney U test [24,25]) and parametric (*t*-test [26]) statistical methods for comparing the vectors *a* and *b* in each of the d = 99 variables. The correlations of the absolute values of $\left|FC\right|$ with the values of $-\log_{10}\left(p\right)\;$ were assessed by calculating Spearman’s ρ [27].

#### 2.4.4. Evaluation of FC Calculation Method Dependency in Biomedical Data

Fold-change calculations are widely available in the biomedical literature. Therefore, for the present reassessment of fold-change calculation methods, the analysis was limited to an extended multi-omics data set (Data set # 3; Table 2). It originates from recent rheumatologic research and consists of an ongoing omics study of a cohort clinically described in [28]. This cross-sectional study of patients with rheumatic diseases was conducted in accordance with the Declaration of Helsinki on Biomedical Research Involving Human Subjects and was approved by the Ethics Committee of the Medical Faculty of the Goethe University, Frankfurt am Main, Germany (approval number 19-492_5). Informed written consent was obtained from each participant. For the present analysis, a subset of cases consisting of n = 95 patients with psoriatic arthritis and n = 50 healthy controls has been used. The omics assessments included d = 680 plasma concentrations of d = 328 proteins from an inflammatory panel and d = 352 lipid markers.

## 3. Results

### 3.1. Reporting Styles of Fold-Change Calculation in Biomedical Publications

The search of PubMed on 23 March 2024, using the query “(“fold change” NOT (review[PT])” returned 10,978 results (Figure 1). However, the true prevalence of fold-change reporting is likely much higher, as researchers often employ fold-change calculations and visualizations without explicitly using the term in titles, keywords, or abstracts. The number of publications per year has been increasing steadily since the turn of the century. An analysis of the context in which FC reporting of omics research results is most common revealed five main biomedical topics (pharmacological research, cancer, infection, immune processes, metabolism) and seven variants of omics research (with a recent publication each: proteomics [29], metabolomics [30], transcriptomics [31], genomics [32], lipidomics [33], multiomics [34], toxicogenomics [35]; Figure 1).

The exact calculation of fold-change (FC) values is rarely reported in the literature. A review of over 200 papers found that only about 5% mentioned the FC calculation method, often in the context of informatics approaches to differential expression analysis rather than the use of FC in reporting biomedical findings. Among the few relevant papers, some used the arithmetic mean [12,36], while others mentioned log transformation, hinting at the use of the geometric mean [37], though this was rarely stated explicitly. Additionally, FC is sometimes calculated from pre-transformed data, such as in the $2^{-\Delta \Delta CP}$ method [38] as a standard in polymerase chain reaction (PCR) data analysis [39].

### 3.2. Role of the Data Distribution for the Correct Calculation of FC

**Figure 2:**
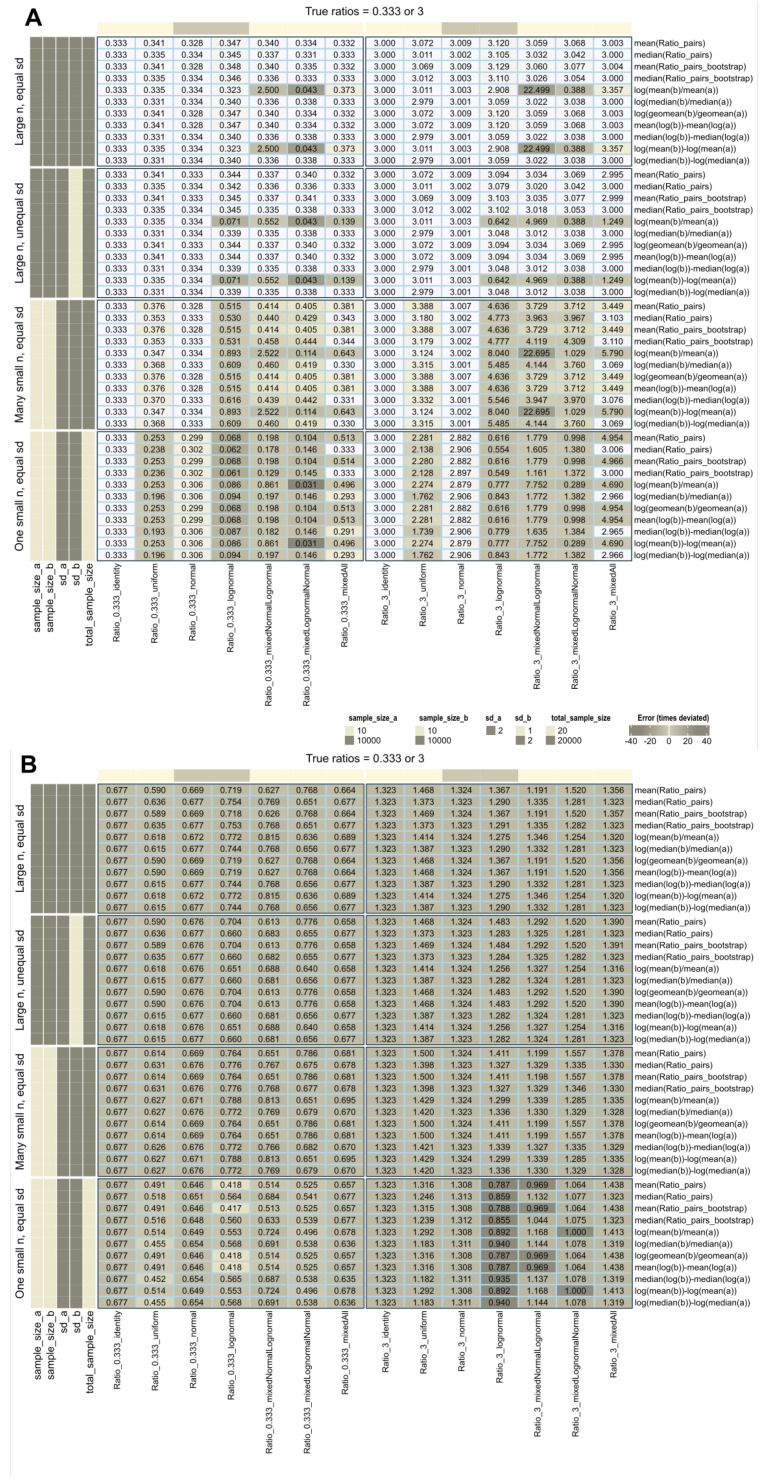
Recovery of fold-change values used to generate artificial data (data set #1) with different distributions (identity, uniform, normal, and lognormal), including mixed distributions such as “mixed”, i.e., a mixture of all four distributions mentioned, or “mixedNormalLognormal” (treatment: normal, reference: lognormal) or “mixedlognormalNormal” (treatment: lognormal, reference: normal). The fold-change calculations are shown on the right and were performed using different methods as specified in Table 1. (**A**) calculations without further data transformation. (**B**) All data were again log-transformed, regardless of their original distribution. The cells are colored according to the errors in fold-change estimates quantified by Equation (6), with darker colors indicating higher absolute errors.

When the data distribution was normal, identity, or uniform and the sample size was large (n = 10,000), all calculation methods accurately recovered the true treatment-to-reference ratios (Figure 2A). However, when log-normal data were included, recovery was confounded by various conditions: most methods succeeded when the standard deviations of the treatment and reference groups were equal, except for the ratio of arithmetic means when the treatment and reference distributions were different. In contrast, all methods except the ratio of arithmetic means were robust to unequal variances.

Recovery deteriorated drastically with small sample sizes, especially with log-normal data, and none of the methods provided accurate results. Repeating the experiments with small sample sizes slightly improved recovery for normal or identity distributions.

The intuitive use of fold-change assumes absolute expression levels. However, a pitfall arises when using pre-transformed data from standard workflows without realizing it. Applying additional log-transformations means log transforming already log-transformed data (Figure 2B). Rankings of variables may be preserved, but calculated fold changes no longer represent “times expression”, only arbitrary numbers, possibly better expressed as “times signal” as a more neutral description. When comparing studies or multi-omics data from different workflows, changes in fold-change magnitude, interpretation, and comparability become relevant.

### 3.3. Role of Variance Equality for the Correct Calculation of FC

**Figure 3:**
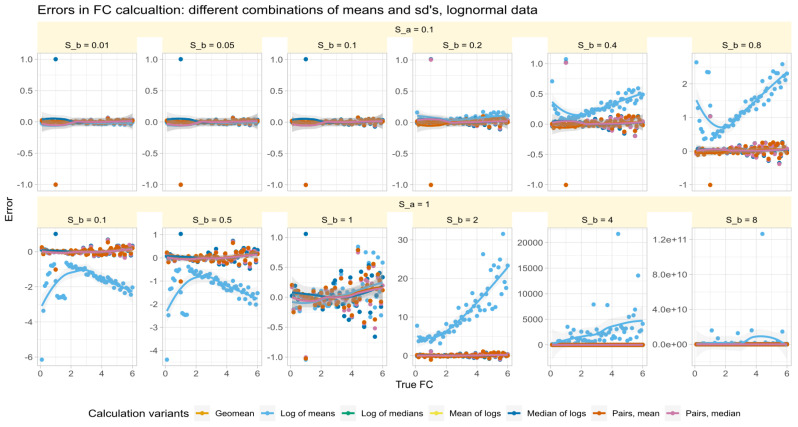
Errors of fold-change recovery (Equation (6)) from synthetic data (data set #2) generated along a range of fold-change values of $FC=\left[0.1,\ldots ,6\right]$ with standard deviation of reference of $s_{a}=0.1$ (upper line of panels) and $s_{a}=1$ (lower line of panels) and standard deviations of the treatment subgroup ($s_{b}$) at ratios $\frac{s_{b}}{s_{a}}=\left[0.1,0.5,1,2,4,8\right]$ (panels from left to right). The trends of the relations are shown as linear regression lines with 95% confidence intervals for the fits. The fold-change calculations were performed using different methods as specified in Table 1.

The errors in test/reference ratios calculated by major methods in Data Set #2 were analyzed for log-normal data with varying standard deviations. The results (Figure 3) showed that all methods except those based on arithmetic means made small errors of about 0.1 times more or less than the true factor. The arithmetic mean-based method had varying effects depending on the fold change and standard deviation of the test relative to the reference.

Specifically, when $s_{b}$ and $s_{a}$ were not equal, the error reversed direction at a value of FC=1. That is, when $s_{b}<s_{a}$ and FC<1, the arithmetic mean-based method overestimated the value of FC, was close to zero when $s_{b}∼s_{a}$, and underestimated the error when $s_{b}>s_{a}$. Thus, the success of correctly estimating FC by arithmetic means depended first on the similarity of the standard deviations. This recalls a precondition of the FC calculation mentioned in [2], namely that FC can be considered a special case when the variances of all genes are equal.

### 3.4. Relationship between Calculated Fold Change and Statistical Significance

Intuitively, higher fold changes tend to be associated with higher statistical significance because larger expression differences between conditions are more likely to be statistically significant, assuming consistent variability within each condition. However, deviations from this intuitive expectation can occur due to high and different variability within and between conditions, leading to lower significance despite large fold changes.

**Figure 4:**
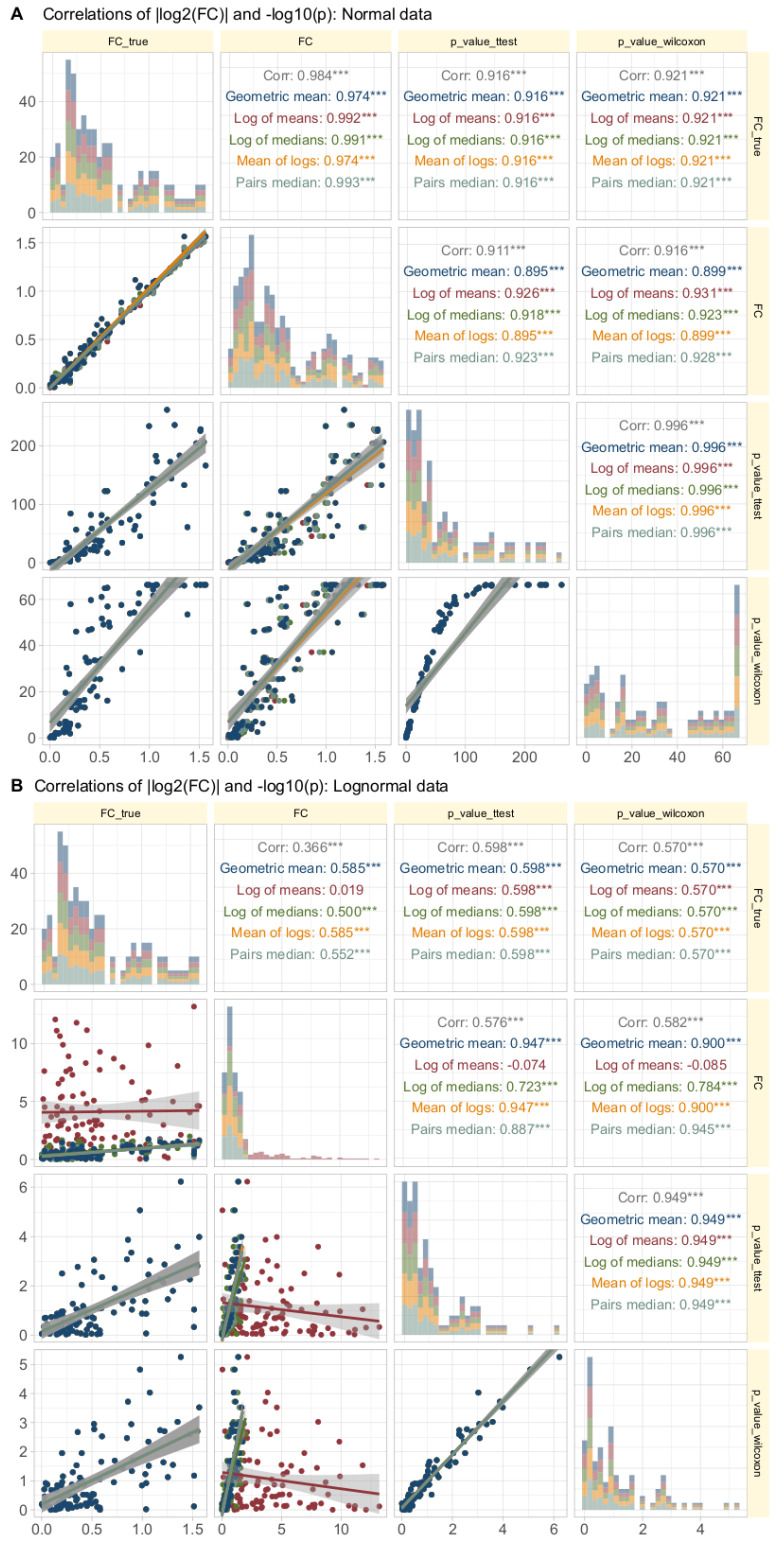
Correlations of |${log}_{2}\left(FC\right)$| and ${-log}_{10}\left(p\right)$ in synthetic data (data set #3) with normal (panel **A** at the top) or log-normal (panel **B**, bottom) distribution. Each d = 99 treatment and reference data sets were generated by randomly assigning fold-change values and treatment ($s_{b}$) and reference ($s_{a}$) standard deviations from predefined ranges. The trends of the relations are shown as linear regression lines with 95% confidence intervals for the fits. The diagonal shows stacked histograms of the distributions of the respective values. “$FC_{true}$” denotes the absolute value of the log2 treatment/reference ratio used during data generation, i.e., |log(FC)|, “FC” denotes the same for the value calculated according to different equations. The upper right triangle shows the Spearman’s correlation coefficient ρ, with stars indicating the significance level (***: p<0.001). The fold-change calculations were performed using different methods, as specified in Table 1.

**Figure 5:**
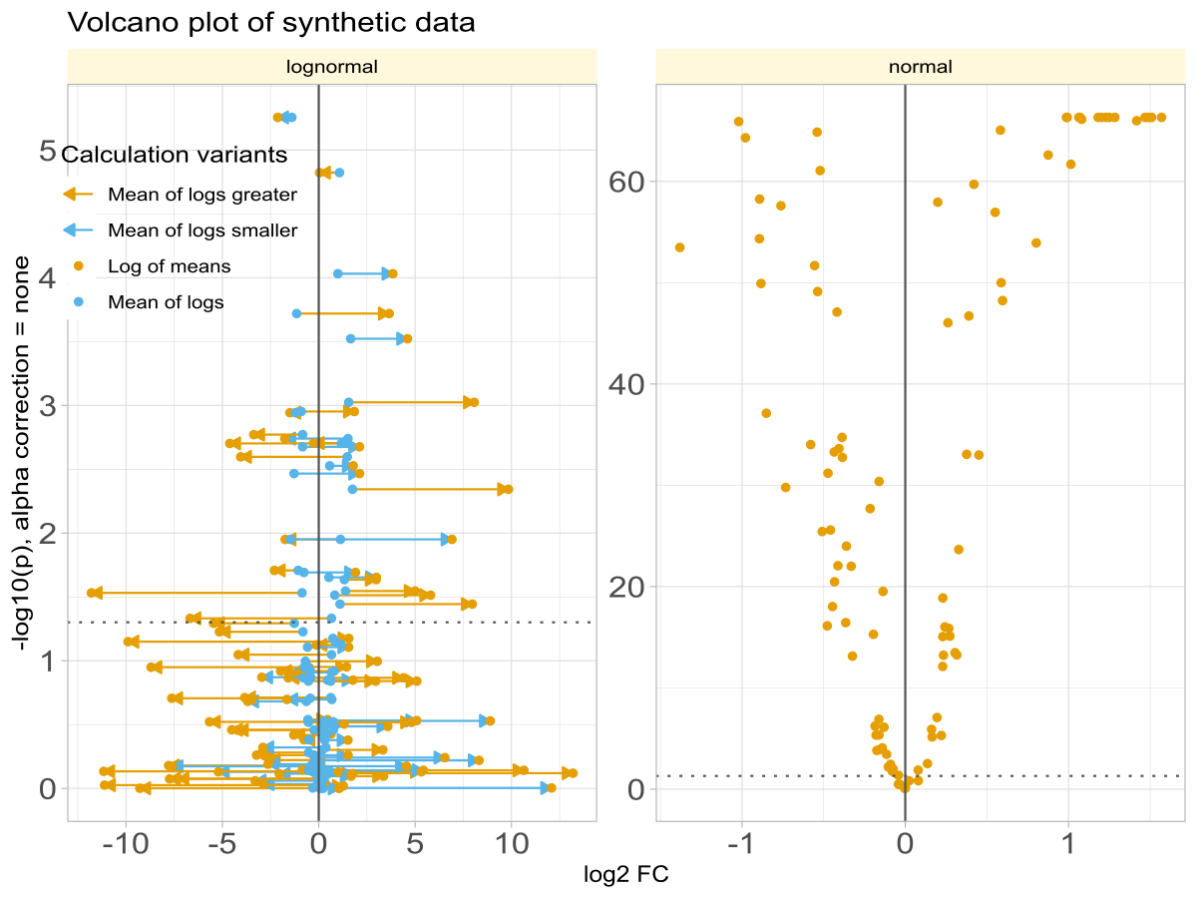
Volcano plots of synthetic data (data set #3) with log-normal (**left** panel) or normal (**right** panel) distribution, obtained when fold changes were estimated using either “log of means”: calculation using the arithmetic mean as the definition of the expected or average value of each subgroup (Equation (3)) or the “mean of logs”: calculation using the arithmetic mean of the logs of treatment and reference as the definition of the expected or average value of each subgroup. The lines connect the points on the diagram that represent the same variables with FC calculated by either method. The ordinate displays $-log_{10}\left({p\_\mathrm{values}}_{Wilcoxon\;test}\;\right)$ without α-correction.

Data Set #3 tested the robustness of fold change (FC) calculation by combining different fold changes with varying standard deviations in normally and log-normally distributed data. In normal distributed data, the choice of the FC calculation method was nevertheless irrelevant to the results (Figure 4A). The obtained values of FC correlated with the true values of FC and with the statistical significance of the group comparisons, and the correlations were quite similar regardless of the FC calculation method. The picture changed somewhat for log-normal data (Figure 4B), where it became clear that calculating FC using the logarithm of the mean was associated with a higher risk of inaccuracy than the alternatives. This was evident in the volcano plot (Figure 5), where extreme cases showed crossover from downregulation to upregulation or vice versa.

### 3.5. FC Calculation Method Dependency in Biomedical Data

**Figure 6:**
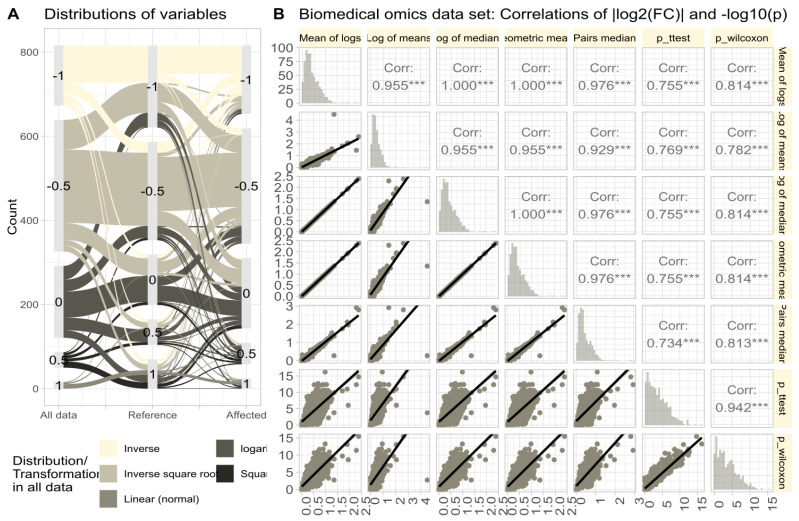
Untransformed real-life omics data (proteomics, lipidomics; data set #4). (**A**) Distribution of variables according to the λ of the Box-Cox analysis. Sankey plot [42] showing the distribution of variables (i) in the complete data and separately for (ii) reference and (iii) treatment subgroups. (**B**) Correlations of |${log}_{2}\left(FC\right)$| and ${-log}_{10}\left(p\right)$ in untransformed real-life omics data (proteomics, lipidomics) after complete permutation of the 2D-matrix in x and y direction. The trends of the relations are shown as linear regression lines with 95% confidence intervals of the fits. The diagonal shows stacked histograms of the distributions of the respective values. The upper right triangle shows the Spearman’s correlation coefficient ρ with stars indicating the significance level (***: p<0.001). The fold-change calculations were performed using different methods, as specified in Table 1.

**Figure 7:**
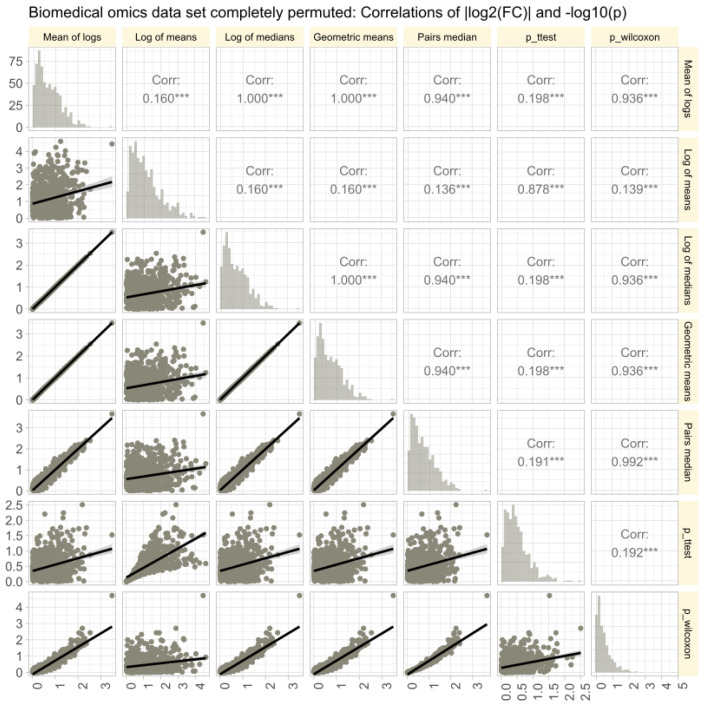
Correlations of of |${log}_{2}\left(FC\right)$| and ${-log}_{10}\left(p\right)$ in untransformed real-life omics data (proteomics, lipidomics) after complete permutation of the 2D-matrix in x and y direction. The trends of the relations are shown as linear regression lines with 95% confidence intervals of the fits. The diagonal shows stacked histograms of the distributions of the respective values. The upper right triangle shows the Spearman’s correlation coefficient ρ with stars indicating the significance level (***: p<0.001). The fold-change calculations were performed using different methods, as specified in Table 1.

The omics data set #4 from rheumatology research showed diverse distributions, with only 14.3% of variables normally distributed in the raw data (Figure 6A). Log-transforming the data increased normality to 48.1%. Therefore, the Box-Cox transformation [40] was used to align the obtained values of λ with the steps of Tukey’s ladder of power [41]. The calculation of FC using different methods (logarithm of means, means of logs, etc.; Figure 6B) yielded similar results, with high correlations between FC values and statistical significances in parametric and non-parametric tests. Further stressing of the calculations by complete permutation of the entire data matrices in the x- and y-directions resulted in highly skewed data (skewness = 23.4, kurtosis = 837.6). Then, the correlations between methods decreased, and the pairwise method showed the strongest correlation with nonparametric test results (Figure 7).

## 4. Discussion

Fold-change calculation is widely used to describe the effect of a treatment on a measurement or group differences. Despite its ease of calculation, the exact method used is often not reported. Although different methods exist, they appear to give similar results. However, a re-evaluation of these methods found that one method, using the arithmetic mean, is less robust to anomalies in the data distribution and can result in incorrect fold-change values more frequently than others. Unfortunately, this is often cited as the standard method for FC calculation [7,43].

The present experiments highlight the importance of sample size, distribution, and variance for accurate fold-change calculation. For small sample sizes (e.g., n = 10), none of the methods could accurately reproduce true ratios. This limitation has been addressed in studies on sample size calculation for differential expression [44,45], but variance estimation from small samples can be generally unreliable [2]. In addition, if the FC calculation is considered a special case when the variances of all genes are equal, the calculation via the supposed standard equation can go wrong.

The present biomedical data showed different distributions of the test and reference data subsets (Figure 6A), albeit to a degree that induced moderate consequences, but nevertheless show the possibility of such a scenario. The arithmetic mean fold-change calculation was most sensitive to violations of assumptions, and the logarithmic calculation performed worst when data were log-normally distributed. In settings with unequal distributions, the arithmetic mean calculation diverged from true values, while alternative equations provided accurate results and maintained correlation with statistical significance.

In the present experiments, different mathematically identical calculation equation variants were used to improve clarity and serve as internal validation. However, we could not reproduce the distinction between ${FC}_{ratio}$ and ${FC}_{difference}$ made in previous work [7], as it seemed to interpret a difference as a ratio [46]. Here, we use fold change exclusively as a ratio. Evaluations focused on calculating numerical fold-change values without further refinement of up- or downregulation [47]. Comprehensive assessments highlight the need for more precise methods, especially for single-cell RNA-seq data [48], as the chosen statistical or fold change cutoff can provide multiple answers for microarray analysis [49].

Finally, the recommendation to use the median or geometric mean to quantify the expected value or group mean must be combined with the warning that even these values are not insensitive to unusual data constellations. The geometric mean is usually appropriate for log-normal data. The median is more general but can also be misleading. For example, in the analysis of right-skewed distributions observed in the analysis of social dynamics, the median can often be more misleading than the mean [50], and such scenarios are also not excluded in biomedical data. Above all, this simple example emphasizes that careful data exploration during preprocessing, including the adequate visualization of raw data [51], as an essential part of the omics data analysis workflow, cannot be replaced by an unquestioned standard procedure rigidly applied to the data at hand.

## 5. Conclusions

Fold-change reporting is widely used to summarize differential expression patterns, but the exact calculation method is often unclear. Different equations can produce different results, especially when data distributions are unequal. To ensure accurate interpretation and reproducibility, it is crucial to use methods less sensitive to data distribution and accurately report the calculation methods used [52]. The inferior arithmetic mean-based method is often perceived as the standard, despite mathematically different equations being possible that mainly differ in the estimation of the expected value. In conclusion, the choice of fold-change calculation method can significantly influence the interpretation of results and subsequent decision-making processes in biomedical research. Adopting less vulnerable methods and transparent reporting is a reasonable practice to ensure correct interpretation and reproducibility.

## Figures and Tables

**Table 1:** Calculation of log ratios between treatment/test (*b*) and reference (*a*). The left column gives the short names with equations indicated; the middle column gives the short names or descriptions used throughout the report, including in the figures; and the right column gives the calculations performed. The right column refers to the calculation method for the corresponding equation number in this report.

Definition of Expected Value	Equation Name	Short Name	Calculation	Equation #
Mean	log(mean(b)/mean(a))	Log of means	$logRatio={log}_{2}\left(\bar{b_{i}}/\bar{a_{i}}\right)$	Equation (3)
Mean	log(mean(b))-log(mean(a))	Log of means	$logRatio={log}_{2}\left(\bar{b_{i}}\right)-{log}_{2}\left(\bar{a_{i}}\right)$	Equation (3)
Median	log(median(b)/median(a))	Log of medians	$logRatio={log}_{2}\left(\tilde{b_{i}}/\tilde{a_{i}}\right)$	Like Equation (3) but median
Median	log(median(b))-log(median(a))	Log of medians	$logRatio={log}_{2}\left(\tilde{b_{i}}\right)-{log}_{2}\left(\tilde{a_{i}}\right)$	Like Equation (3) but median
Geometric mean	log(geomean(b)/geomean(a))	Geometric mean	${logRatio=log}_{2}\left(\frac{2^{\bar{\log_{2}b_{i}\;}}}{2^{\bar{\log_{2}a_{i}\;}}}\right)$	Equation (4)
Geometric mean	mean(log(b))-mean(log(a))	Mean of logs	$logRatio=\bar{\log_{2}b_{i}\;}-\bar{\log_{2}a_{i}\;}$	Equation (4)
Mean of logs	median(log(b))-median(log(a))	Median of logs	$logRatio=\tilde{\log_{2}b_{i}\;}-\tilde{\log_{2}a_{i}\;}$	Like Equation (4) but median
Paired fold change combinations	mean(Ratio_pairs)	Pairs mean	$logRatio=\bar{{log}_{2}\left(\frac{b_{i}}{a_{j}}\right)}\;for\;\left(\left(a_{j},b_{i}\right)\;\epsilon \;{pairs}_{ab}\right)$	Equation (5)
Paired fold change combinations	median(Ratio_pairs)	Pairs median	$logRatio=\tilde{{log}_{2}\left(\frac{b_{i}}{a_{j}}\right)}\;for\;\left(\left(a_{j},b_{i}\right)\;\epsilon \;{pairs}_{ab}\right)$	Like Equation (5) but median
Paired fold change combinations	mean(Ratio_pairs_bootstrap)	Pairs mean bootstrap	$logRatio=\bar{{log}_{2}\left(\frac{b_{i}^{*}}{a_{j}^{*}}\right)}\;for\;\left(\left(a_{j},b_{i}\right)\;\epsilon \;{bootstrapped\;pairs}_{ab}^{*}\right)$	Like Equation (5) but bootstrapped pairs
Paired fold change combinations	mean(Ratio_pairs_bootstrap)	Pairs median boostrap	$logRatio=\tilde{{log}_{2}\left(\frac{b_{i}^{*}}{a_{j}^{*}}\right)}\;for\;\left(\left(a_{j},b_{i}\right)\;\epsilon \;{bootstrapped\;pairs}_{ab}^{*}\right)$	Like Equation (5) but bootstrapped pairs

**Table 2:** Artificial data sets were created to assess certain effects of data distribution on the correct recovery of FC values by different calculation methods. All data sets contained vectors *a* and *b*, with vector *a* serving as the reference and the values of vector *b* being FC times the values of vector *a*. That is, vector *a* had a mean, if applicable, of $m_{a}=m$, a standard deviation, if applicable, of $s_{a}$, and a sample size of $n_{a}$. Vector *b* had a mean of $m_{b}=FC•m_{a}$, a standard deviation of $s_{b}$, and a sample size of $n_{b}$. $U_{1}$ and $U_{2}$ denote independent uniform distributions, and *N* denotes the normal distribution.

Data Set	Distribution	Generation
Data set #1	Identity	$a\;=\;\underset{n_{a}}{\underset{︸}{m_{a},\;m_{a},\;\ldots ,\;m_{a}}}$ $b\;=FC•\;\underset{n_{b}}{\underset{︸}{m_{a},\;m_{a},\;\ldots ,\;m_{a}}}$
	Uniform	${a=\;U}_{1}\left(n_{a},0,1\right)•m_{a}$ ${b=\;U}_{2}\left(n_{b},0,1\right)•m_{a}•FC$
	Normal	$a=a_{1},a_{2},\ldots ,a_{n_{a}}\;∼\;N\left(m_{a},s_{a}\right)$ $b=b_{1},b_{2},\ldots ,b_{n_{b}}\;∼\;N\left({FC•m}_{a},s_{b}\right)$
	Log-normal	$a=a_{1},a_{2},\ldots ,a_{n_{a}}\;∼\;LogNormal\left(\log\left(m_{a}\right)\;,s_{a}\right)$ $b=b_{1},b_{2},\ldots ,b_{n_{b}}\;∼\;LogNormal\left(\log\left(FC\right)\;+\log\left(m_{a}\right)\;,s_{b}\right)$
	mixedNormalLognormal	$a=a_{1},a_{2},\ldots ,a_{n_{a}}\;∼\;N\left(m_{a},s_{a}\right)$ $b=b_{1},b_{2},\ldots ,b_{n_{b}}\;∼\;LogNormal\left(\log\left(FC\right)\;+\log\left(m_{a}\right)\;,s_{b}\right)$
	mixedLogormalNormal	$a=a_{1},a_{2},\ldots ,a_{n_{a}}\;∼\;LogNormal\left(\log\left(m_{a}\right)\;,s_{a}\right)$ $b=b_{1},b_{2},\ldots ,b_{n_{b}}\;∼\;N\left({FC•m}_{a},s_{b}\right)$
	Mixed	$a=\;U_{1}\underset{n_{a}}{\underset{︸}{\left\{\left\{a_{Identity}\right\},\left\{a_{\mathrm{Uniform}}\right\},\left\{a_{\mathrm{Normal}}\right\},\left\{a_{\mathrm{Log}-\mathrm{normal}}\right\}\right\}}}$ $b=\;U_{2}\underset{n_{b}}{\underset{︸}{\left\{\left\{b_{Identity}\right\},\left\{b_{\mathrm{Uniform}}\right\},\left\{b_{\mathrm{Normal}}\right\},\left\{b_{\mathrm{Log}-\mathrm{normal}}\right\}\right\}}}$
Data set #2	Log-normal	$a=a_{1},a_{2},\ldots ,a_{n_{a}}\;∼\;LogNormal\left(\log\left(m_{a}\right)\;,s_{a}\right)$ $b=b_{1},b_{2},\ldots ,b_{n_{b}}\;∼\;LogNormal\left(\log\left(FC\right)\;+\log\left(m_{a}\right)\;,s_{b}\right)$
Data set #3	Normal	$a\;∼N\left(m,s_{a}\right)$ $b\;∼N\left(FC•m,s_{b}\right)$
	Log-normal	$a\;∼LogNormal\left(\log\left(m\right)\;,s_{a}\right)$ $b\;∼\;LogNormal\left(\log\left(FC\right)\;+\log\left(m\right)\;,s_{b}\right)$

## Data Availability

The biomedical data set used in the experiments in this report is available from the first author upon reasonable request and subject to approval by the appropriate ethics committee. The artificial data sets’ generation rules are precisely described in the report.
